# Zebrafish Patient-Derived Xenograft Model to Predict Treatment Outcomes of Colorectal Cancer Patients

**DOI:** 10.3390/biomedicines10071474

**Published:** 2022-06-22

**Authors:** Gregorio Di Franco, Alice Usai, Margherita Piccardi, Perla Cateni, Matteo Palmeri, Luca Emanuele Pollina, Raffaele Gaeta, Federica Marmorino, Chiara Cremolini, Luciana Dente, Alessandro Massolo, Vittoria Raffa, Luca Morelli

**Affiliations:** 1 General Surgery Unit, Department of Translational Research and New Technologies in Medicine and Surgery, University of Pisa, Via Paradisa 2, 56124 Pisa, Italy; gregorio.difranco@med.unipi.it (G.D.F.); matteo.palmeri@med.unipi.it (M.P.); 2 Department of Biology, University of Pisa, S.S. 12 Abetone e Brennero 4, 56127 Pisa, Italy; alice.usai@biologia.unipi.it (A.U.); margherita.piccardi@phd.unipi.it (M.P.); p.cateni@studenti.unipi.it (P.C.); luciana.dente@unipi.it (L.D.); alessandro.massolo@unipi.it (A.M.); vittoria.raffa@unipi.it (V.R.); 3 Department of Surgical, Medical, Molecular Pathology and Critical Area, Division of Surgical Pathology, University of Pisa, Via Paradisa 2, 56124 Pisa, Italy; l.pollina@ao-pisa.toscana.it (L.E.P.); raffaele.gaeta@med.unipi.it (R.G.); 4 Unit of Medical Oncology 2, Azienda Ospedaliero-Universitaria Pisana, Via Roma 67, 56126 Pisa, Italy; federica.marmorino@gmail.com (F.M.); chiara.cremolini@unipi.it (C.C.); 5 Department of Translational Research and New Technology in Medicine and Surgery, University of Pisa, 56124 Pisa, Italy

**Keywords:** zebrafish avatar, chemosensitivity, preclinical model, colorectal cancer, personalized medicine

## Abstract

The use of zebrafish embryos for personalized medicine has become increasingly popular. We present a co-clinical trial aiming to evaluate the use of zPDX (zebrafish Patient-Derived Xenografts) in predicting the response to chemotherapy regimens used for colorectal cancer patients. zPDXs are generated by xenografting tumor tissues in two days post-fertilization zebrafish embryos. zPDXs were exposed to chemotherapy regimens (5-FU, FOLFIRI, FOLFOX, FOLFOXIRI) for 48 h. We used a linear mixed effect model to evaluate the zPDX-specific response to treatments showing for 4/36 zPDXs (11%), a statistically significant reduction of tumor size compared to controls. We used the RECIST criteria to compare the outcome of each patient after chemotherapy with the objective response of its own zPDX model. Of the 36 patients enrolled, 8 metastatic colorectal cancer (mCRC), response rate after first-line therapy, and the zPDX chemosensitivity profile were available. Of eight mCRC patients, five achieved a partial response and three had a stable disease. In 6/8 (75%) we registered a concordance between the response of the patient and the outcomes reported in the corresponding zPDX. Our results provide evidence that the zPDX model can reflect the outcome in mCRC patients, opening a new frontier to personalized medicine.

## 1. Introduction

Colorectal cancer was estimated as the third most common malignancy and the second cancer-causing patient death in 2020 [1]. Several sub-types of colorectal cancer are described, and each patient has a heterogeneous cancer cell population, with the consequence that each individual patient responds differently from others to the conventional therapies (chemotherapy and/or radiotherapy) [2]. In the last years, the interest in precision medicine has increased enormously, with the intent to customize the treatment based on each patient’s tumor characteristics, especially on the biology of its own tumor. With this intent, preclinical studies need to be set up to assess the drugs’ effectiveness. In fact, the apparently simple question “Which chemotherapy is the best option?” is harder to address with certainty [3]. In this setting, starting from patient-derived tissues or cells, it is possible to obtain in vitro and in vivo avatars. The first ones consist of spheroids obtained by tumor tissue dissociation; explants, that are not dissociated and retain their tissue structure; and organoids, obtained from adult stem cells [4]. Meanwhile, the Patient-Derived Xenograft (PDX) in vivo models are emerging as an alternative approach for precision medicine for patients with solid tumors [5]. PDXs are obtained by implanting tumor tissue or primary cells taken directly from the patient’s tumor into animal models, with the advantage of maintaining the heterogeneity of the original tumor [6,7,8,9] and using them in drug efficacy studies. These include mouse or zebrafish PDX models. In the last decade, zebrafish Patient-Derived Xenograft (zPDX) models have increased in popularity, helping to solve many of the limitations of traditional cancer models [10,11,12]. Different studies have shown that given the short-term drug exposure required, both larval and adult zPDXs could address the in vivo drug response in a reasonable time frame essential for their translation to the clinic [10,12,13]. In this scenario, the zPDX approach may show the response of the tumor biopsy samples to drugs, introducing the model in the field of precision cancer therapy [14]. Therefore, our aim is to evaluate the possibility to use zPDXs for patients with colorectal cancer. After the encouraging results that we have already reported about the first zebrafish larval co-clinical trial (XenoZ, NCT03668418) for patients with pancreatic ductal adenocarcinoma (PDAC) [15,16], we are now presenting the results obtained with the zPDX of patients that underwent surgical resection for colorectal cancer. Instead of using the procedure of isolated cancer cells xenografts, we decided to use the approach in which pieces of the patient’s tumor tissue are directly xenotransplanted into zebrafish embryos, taking advantage that they have not yet developed an adaptive immune response. Therefore, we firstly report the development of zPDXs model of colorectal cancer with the ability to predict the response to chemotherapy according to the Response Evaluation Criteria in Solid Tumors (RECIST). This allows to assess the patients’ response profiles, with the potential implications in everyday clinical practice for oncologists during their clinical decisions.

## 2. Materials and Methods

### 2.1. Human Trial

The observational prospective co-clinical trial (XenoZ, NCT03668418) was approved by the local ethics committee (prot. n. 70213) and conducted in accordance with the guidelines of the European Network of Research Ethics Committees. All donors were enrolled after their informed consent and underwent surgical excision of colon cancer at the University of Pisa (Italy). The specimen was analyzed by the pathologist, and a fragment of the tumor was taken for the establishment of zPDX.

Preoperative assessments were recorded including diagnosis, age, gender, and body mass index (BMI). Intraoperative data included type of surgical procedure, and eventually if there were complications to take a fragment of the tumor for research purpose. Histological data included the following: the histological type of the tumor, grade of differentiation, tumor dimension, number of harvested lymph nodes, and number of metastatic lymph nodes. Patients were staged according to the T and N definitions proposed for the American Joint Committee on Cancer 8th edition [17].

### 2.2. Zebrafish Housing and Husbandry

Husbandry procedures of zebrafish were conducted in conformity with the EU Directive 2010/63 and authorized by the local animal welfare committee (authorization n. 99/2012-A, 19/04/2012; authorization for zebrafish breeding for scientific purposes released by the “Comune di Pisa” DN-16/43, 19 January 2015). Zebrafish were housed in facility at the temperature of 28 °C, pH 7.4–7.5, and conductivity 500–800 µS. Temperature, pH, and conductivity were automatically adjusted, whereas the nitrogen compounds were monitored weekly. We collected fertilized eggs from natural mating of AB wild-type zebrafish and the embryos were incubated in petri dish at 28 °C until they were used for experimental purpose. Before the xenotransplantation procedure, zebrafish embryos were exposed in the anesthetic MS 222 at a concentration of 0.16 mg/mL.

### 2.3. Patient Material Processing and zPDX Model

The applied methods were previously described in detail by our team [12]. Briefly, a fragment of a patient-derived tumor was obtained from colon resection at the Division of Surgical Pathology (University of Pisa, Pisa, Italy) and screened by the pathologist. The material was stored in collection medium (RPMI supplemented with 100 U/mL penicillin, 100 µg/mL streptomycin, and 2.5 µg/mL amphotericin) at 4 °C and processed within 16 h after the resection. Using scalpels, the tissue was minced into fragments of 1–3 mm^3^, and then chopped using the McIlwain tissue chopper (Campden Instruments LTD, Leicestershire, UK). The pieces were stained with 20 µg/mL CellTracker^TM^ CM-Dil (Life Technologies Corporation, Eugene, OR, USA) in D-PBS and placed for 30 min in a water bath at 37 °C. Tissue pieces were then washed by D-PBS and centrifuged at 300× *g* for 3 min, three times. The pieces were resuspended in D-PBS supplemented with 10% FBS (Life Technologies Limited, Paisley, UK). After two days post fertilization (dpf), AB wild-type embryos were used to establish zPDX. The embryos were placed on one side in 1% agarose disks. The xenografts were manually performed by inoculating a fragment into the perivitelline space of *n* = 90 zebrafish embryos. Specifically, each tumor fragment was picked up and placed on top of the perivitelline space using a Dumont No. 5 fine forceps, then the microinjection was performed by using a borosilicate glass microneedle heat pulled. All procedures were performed under sterile conditions. After transplantation, embryos were incubated for 2 h at 35 °C.

### 2.4. Zpdx Treatment

Embryos were checked for positive xenografts, then they were randomly distributed one embryo per well of a 48 multi-well and equally assigned to one of the five groups (four therapeutic options and one control group, *n* ≥ 10 of embryos/group). Images were acquired at 2 h post injection (hpi) as experimental starting point and at 48 hpi as experimental end point by a fluorescence microscope (Nikon, TE2000-U, Tokyo, Japan). As previously described [12] after the image acquisition at 2 hpi, fresh drugs were administered at the equivalent dose (ED = 5) and embryos were incubated at 35 °C. The chemotherapy regimens tested were: 5-Fluorouracil (5-FU) in place of Capecitabine that administered to humans is metabolized in 5-FU, 5-Fluorouracil + Folinic acid + Oxaliplatin (FOLOFOX), 5-Fluorouracil + Folinic acid + Irinotecan (FOLFIRI), and 5-Fluorouracil + Folinic acid + Oxaliplatin + Irinotecan (FOLFOXIRI). The drugs were diluted in zPDX medium (E3 supplemented with 100 U/mL penicillin and 100 µg/mL streptomycin) at the concentrations reported in Table 1 and they were renewed daily. The control group was maintained in zPDX medium.

### 2.5. Data Modelling and Statistical Analysis

As first described by our team for PDAC co-clinical trial [15], a Linear Mixed effect Model (LMM) [18] was adopted to analyze the treatment effects on zPDX tumor and to investigate the zPDX specific response. The xenografted tumor was considered as a spherical object, according to the circularity analysis (see Supplementary Materials). Specifically, from the area analyzed by ImageJ, the equivalent radius (*r*), and consequently the equivalent tumor volume, of each zPDX (%Δ*V*) were estimated with the following formula:

(1) $$\%∆V=\frac{Volume_{48hpi}-Volume_{2hpi}}{Volume_{2hpi}}\;\times \;100$$

In the proposed LMM, %Δ*V* was the outcome variable. To test the mean effect of the chemotherapeutics on zebrafish embryo population, treatments were considered as fixed factors. Treatment was a categorical variable having five categories with four treatment options (5-FU, FOLFOX, FOLFIRI, FOLFOXIRI) and a control group, used as baseline. Whereas, to assess zPDX specific response to treatments, zPDXs were included in the LMM as random effects (36 categories).

The LMM was estimated through a forward stepwise selection [18]: Firstly, the random component of the intercept was included, then the random slope was also considered to evaluate the effect of the treatments in each zPDX. Moreover, as the initial volume (2*hpi*) differed among all zPDX records, we decided to include r^3^ at 2*hpi* as proxy of the initial volume and as a covariate to observe whatever the initial volume could exert an effect on %Δ*V*. During data exploration, the %Δ*V* showed a positive-skew distribution. Consequently, the %Δ*V* was transformed by 10-base logarithm [19] adding the constant value of 110.

To compare levels of fixed effects, post-hoc tests were conducted through the R package *emmeans,* estimating the least square means of each treatment (dummy variables), their standard errors and the 95% confidence intervals (95% CI). The *p*-values were adjusted by Tukey’s HSD method.

We estimated the 95% CI of random effects for each treatment to determine if there was a statistically significant difference between zPDX response and the population mean (fixed effects coefficients). Moreover, we calculated the 95% CI of the %∆*V* predicted by the model, to evaluate the statistical difference of the zPDX treatment response compared to the control.

As described [15], we computed predicted values of %∆*V* and their 95% CIs, adding random effect coefficients to the fitted values of fixed effects. For each zPDX, we contrasted the 95% CIs of predicted %∆*V*s in treatments and controls to evaluate chemotherapeutic ability to reduce the tumoral mass in respect to controls. To test if there was a significant reduction of %∆*V*, we compared 95% CIs of predicted values with 0 on the log scale. A significant decrease of the tumoral mass occurred when the upper limit of 95% CI of predicted %∆*V* was lower than 0 on the log scale.

Moreover, we used the function *ranef* of the lme4 package for R to extract the random effects of the model. Random effects were then used to perform the cluster analysis with Hartigan-Wong K-means algorithm to identify five groups of zPDX who share the same response to treatments pattern.

The random effects were the difference between the zPDX population mean and the random coefficients, representing dissimilarity between the %Δ*V* of each zPDX and the mean of %Δ*V* of zPDX population. To evaluate if the clustered zPDX had a similar response to population mean in all proposed treatments, we matched k-means clusters with 95% CI of random effects.

The statistical analyses were performed using R software version 4.1.1, (https://www.r-project.org (accessed on 10 August 2021)). We also used GraphPad Prism 7 as statistical analysis software. Data analysis was performed by ANOVA, followed by Bonferroni correction or Dunnett’s post-hoc test or t-test. Statistical significance was set to 5%.

### 2.6. Co-Clinical Trial

In the fish trial, the zPDX response during chemotherapy were assessed adapting the RECIST criteria. As previously proposed [12], the efficacy of chemotherapy was evaluated taking the relative stained area at 2-day post-injection (dpi)/2 h post-injection (*hpi*) of the control group as a reference (Figure S1C).

The criteria applied are shown below:

Progressive Disease (PD): increase of ≥20% in the relative stained area at 2 dpi/2 *hpi*.

Stable Disease (SD): decrease of <30% or increase of <20% in the relative stained area at 2 dpi/2 *hpi*.

Partial Response (PR): decrease of ≥30% but <90% in the relative stained area at 2 dpi/2 *hpi*.

Complete Response (CR): decrease ≥90% in the relative stained area at 2 dpi/2 *hpi*.

The response between zPDX and colorectal patients was compared. Patients who had experienced a disease recurrence during the follow-up period and were treated with first-line chemotherapy were included. The follow-up included a total body CT scan performed every three months or in the case of symptoms. The response to chemotherapy in patients was classified according to the RECIST criteria used in the common clinical practice as partial response (PR), stable disease (SD), and progression disease (PD) [20]. The percentages used in the common clinical practice for the classification of the response to chemotherapy are the same that we used in our zPDX model. We compared the response reported in patients with the response reported in PDXs of the same patients for the chemotherapy regimen used as first-line treatment. Patients who were not treated with first-line chemotherapy, or underwent chemotherapy regimen not tested in xenografted zebrafish embryos, were not included into the study. An exclusion criterion was also applied for patients whose zebrafish avatar reported low engraftment. The correlation between human and zPDX response was tested with Kendall rank correlation test performed by R package Kendall.

## 3. Results

From September 2018 to July 2020, 36 patients with colorectal cancer were enrolled. Patients’ characteristics are summarized in Table 2. Of these 36 patients, 14 (38.9%) were female. The mean age was 68.4 ± 12 years (range 32–89) and the mean BMI was 24.4 ± 4.3 kg/m^2^ (range 19.9–32.2). A right hemicolectomy was performed in 15/36 (41.7%) patients, an anterior rectal resection in 9/36 patients (25%), a resection of the left colic flexure in 4/36 (11.1%) patients, a left hemicolectomy in 3/36 (8.3%) patients, an abdominoperineal resection in 2/36 (5.6%) patients, a resection of a local recurrence in 1/36 (2.8%) patient, while in 2/36 (5.6%) the resection of colorectal liver metastases was performed. In no case was there a problem related to the surgery that precluded the possibility of taking a fragment of the tumor from the surgical specimen for the xenotransplantation in the zebrafish embryos. In all cases, it was confirmed the presence of a colic adenocarcinoma or colorectal liver metastasis at the histological examination. A moderately differentiated adenocarcinoma (G2) was reported in 20/36 (55.5%) cases, while a poorly differentiated adenocarcinoma (G3) was reported in 16/36 (45.5%) cases. The mean diameter of the colic neoplasia was 4.7 ± 2.1 cm (range 1.2–10.0). The mean number of harvested lymph nodes was 21.8 ± 11.8 (range 6–64). The presence of positive lymph nodes was documented in 28/36 (77.8%) patients, reporting a mean number of positive lymph nodes of 3.0 ± 2.8 (range 0–9). The presence of tumor budding was reported in 18/36 (50%) patients, while the angioinvasion was reported in 22/36 (61.1%) patients.

### 3.1. Data Modelling

**Figure 1:**
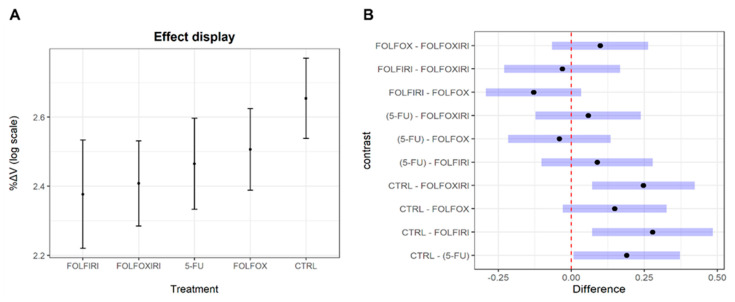
LMM results. (**A**) Effect displays. Treatments are on the x-axis. Dots represent the fixed effect coefficients of %ΔV on log scale estimated by the LMM. The bars are the 95% CIs of fixed effect coefficients. (**B**) Post-hoc test results. Differences of marginal means are on the x-axis and pairwise comparisons between treatments are displayed on the y-axis. Blue bars represent the 95% CIs of marginal means differences estimated through R package emmeans. The dashed line corresponds to a difference of zero between means.

According to the LMM, we observed a significant reduction of %ΔV in all treatments with respect to controls (Figure 1A and Table S1). The post-hoc test confirmed the significant decrease of tumor mass, except for FOLFIRI. In addition, the pairwise comparison did not show a significant difference between treatments (Figure 1B).

Then, calculating the 95% CIs of the difference between fixed and random coefficients, we did not detect a statistical difference between the control group and the overall zPDX mean. Instead, a significant reduction of the tumor volume with respect to mean response of zPDX was observed in 19% (7/36) of zPDX after FOLFOXIRI treatment and in 25% (9/36) after FOLFOX, 5-FU and FOLFIRI treatments (Supplementary Materials, Figure S2).

**Figure 2:**
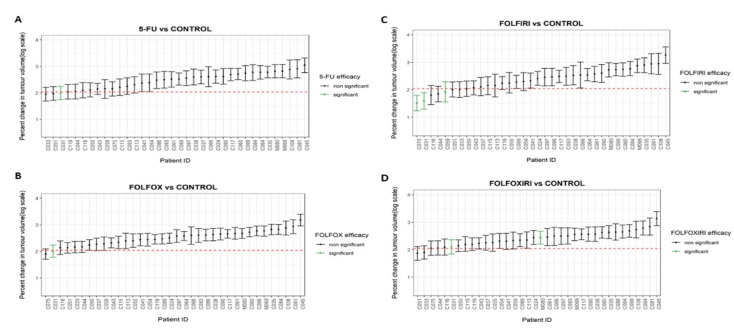
Error bars represent 95% confidence intervals of the %∆V calculated using random-effects model. Subgroup analyses for the treatment efficacy of 5-FU (**A**), FOLFOX (**B**), FOLFIRI (**C**), and FOLFOXIRI (**D**), with respect to each patient control were also conducted. Green 95% confidence intervals represent a statistically significant difference to the control.

From the analysis of 95% CIs predicted %ΔV, it was shown that 5-FU and FOLFOX treatments caused a significant tumor volume to decrease in 1/36 zPDXs (3%), with respect to control (Figure 2A,B). FOLFIRI treatment induced a significant reduction of tumor volume in 3/36 zPDX (8%) (Figure 2C). The efficacy of FOLFOXIRI was significant in 2/36 zPDX (6%) (Figure 2D).

**Figure 3:**
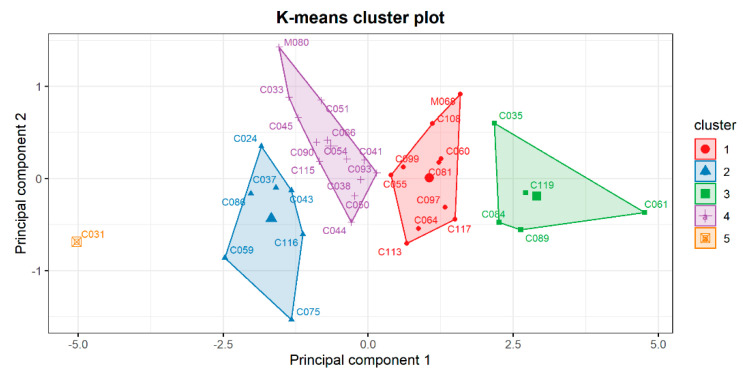
Clustering of colorectal cancer patients according to k-means and principal component analysis.

**Figure 4:**
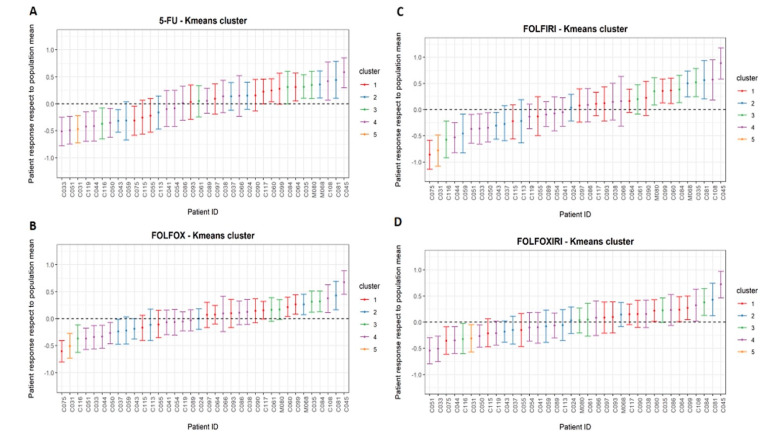
Error bars represented 95% confidence interval of the %∆V for 5-FU (**A**), FOLFOX (**B**), FOLFIRI (**C**), and FOLFOXIRI (**D**) resulted from k-means clustering. The graphs displayed how patients within each chemotherapy treatment were assigned in one of the five cluster groups. The different colors identified different clusters.

Performing the cluster analysis, we initially aimed to identify the four zPDX groups, each one representing zPDX that similarly responded to a specific treatment of the four proposed. However, during preliminary clustering attempts there was always a zPDX (C031) with a trend of tumor volume variation different from other patients, so it tended to cluster alone. Therefore, we decided to add another cluster in the attempt to better represent our data and to clearly describe all patients (Figure 3). In Figure 4 it was reported the cluster to which each patient was assigned for each chemotherapy regimen.

### 3.2. Zebrafish Trial

**Figure 5:**
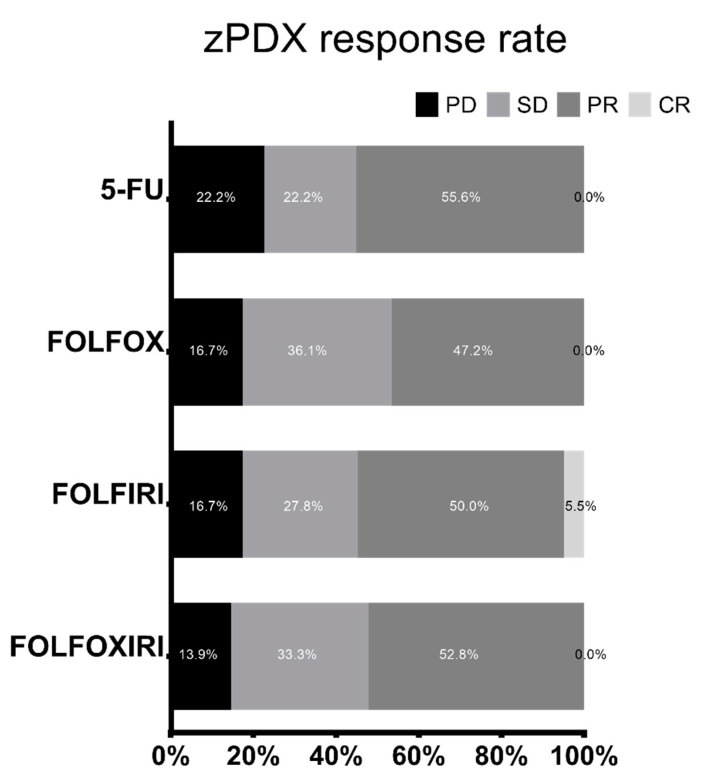
Percentage of progressive disease (PD), stable disease (SD), partial response (PR), and complete response (CR). 5-FU, FOLFOX, FOLFIRI and FOLFOXIRI treatments in zebrafish avatars xenotransplanted with colorectal tumor (n = 36 patient samples analyzed).

For colorectal cancer zPDXs (Figure S1), a PD was observed in 22% of cases treated with 5-FU, in ~17% of cases treated with FOLFOX and with FOLFIRI, and in ~14% of cases treated with FOLFOXIRI. A SD occurred in 22.2% of cases in 5-FU group, in 36.1% cases in FOLFOX group, in ~28% of cases in FOLFIRI group, and 33.3% of cases in FOLFOXIRI group. A PR was detected in ~55% of cases treated with 5-FU, in ~50% of cases treated with FOLFIRI, in ~47% of cases treated with FOLFOX, and in ~58% of cases treated with FOLFOXIRI. In a limited number of patients’ sample (5.6%) we reported a CR, and only to FOLFIRI chemotherapy (Figure 5).

### 3.3. Co-Clinical Trial

**Figure 6:**
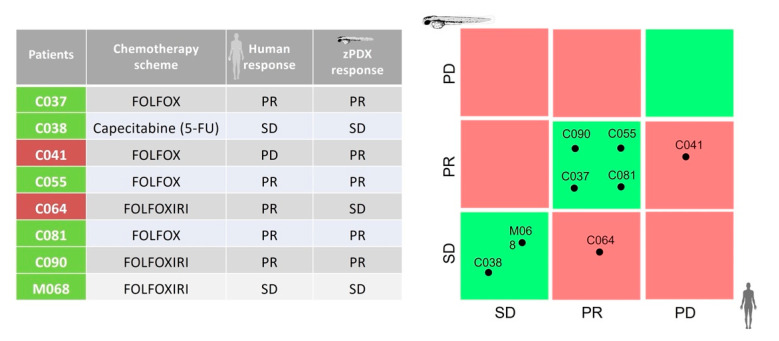
Comparison of the follow-up data with the predicting outcome of the zPDX. PR: partial response, SD: stable disease; PD: progression disease. Capecitabine was orally administered to Patient C038, whereas 5-FU was tested in its zPDX.

Of the 36 patients with colorectal cancer enrolled, 16 patients did not receive adjuvant chemotherapy by choice of the patients or due to the clinical stage of the tumor, 8 patients underwent adjuvant chemotherapy treatment after surgical operation without cancer recurrence, and 12 patients underwent first-line treatment after cancer recurrence. However, in 4 out 12 of metastatic colorectal cancers (mCRC) patients, the treatment is still ongoing. Therefore, only for 8 patients we have both oncological information obtained during the follow-up, in term of response to first-line chemotherapy according to the RECIST criteria and the zPDX chemosensitivity profile of its own tumor xenotransplanted into zebrafish embryos, with the possibility to compare the response reported in zPDXs and the clinical response. Among the eight mCRC patients above-mentioned, five (62.5%) experienced PR and 3 (37.5%) experienced SD (Figure 6). Of these patients, results showed a 75% concordance (6/8 cases) between the clinical response and the efficacy of chemotherapy reported in the zPDXs (Figure 6). The correlation between human and zPDX response tested with Kendall rank correlation test revealed a tau = 0.689 with a 2-sided *p* value = 0.085.

## 4. Discussion

Recent studies analyzing genome cancer profiles reported the presence of tumor heterogeneity not only between cancers (inter-tumor) but also within each cancer (intra-tumor) [10]. Thus, in the current clinical practice it is difficult to securely predict if a patient will likely respond to the chosen chemotherapy treatment. In this scenario, the possibility to test in vitro and in vivo the benefit from chemotherapy regimens is of paramount importance in order to personalize the treatment. However, the in vitro tests could not be used in oncology practice due to their lack of robustness [10]. In the last decade, the use of PDX model in the field of translational research has gained popularity, thanks to the capability of this model to maintain the complexity of the tumor microenvironment and heterogeneity of the original tumor in patient [21]. Currently, the mouse PDX is considered the current gold standard for in vivo assessment of tumor heterogeneity and response to therapy [5]. However, the use of mouse PDX in the clinical practice is not so feasible due to some limitations including the need for expensive immune-permissive strains for the risk of transplant rejection, the need for a large fragment of tumor (about 1 million tumor cells needed), the long time needed to obtain a visible tumor implant (from several weeks to months), and high difficulties to generate mouse xenotransplant models able to metastasize [22]. In contrast to mouse PDX, the use of zebrafish has many advantages, such as the large number of offspring and the short generation time, the rapid development, the simple and inexpensive housing to maintain, the transparency that makes possible to observe the tumor growth without invasive imaging, and the external development of the embryos and their small size [23,24].

Zebrafish are successfully used in different fields of colorectal cancer research, such as in genetic models of cancer to study non-oncogene targets adopting the rapidly developing zebrafish intestinal epithelium as a surrogate tissue for colorectal tumors [25].

However, an important application field of zebrafish embryos is their use as a transplantation cancer model. After the first experiment reported in 2005 [26], experiences with human tumors xenotransplanted into zebrafish has rapidly increased [27], and different xenograft zebrafish models have been developed with the use of various injection sites, developmental stages, and transplanted specimens (i.e., human cell lines, patient-derived primary cancer cells, patient-derived tumor tissue) [13,28,29,30,31].

Experiences with xenotransplantation of human colon cancer cell lines in zebrafish embryos are limited and usually are used to study mechanisms of tumor progression and metastasis process [24,32]. However, the xenotransplantation of commercially available cells lines is limited by its inability to reflect the real response of cancers of each patient [22]. In fact, using cell lines precludes the possibility to take in consideration the heterogeneity of the primary tumor since the process of adaptation induces the selection of clones with a higher proliferative rate than the primary tumor, with the consequent risk that selected cell lines may not be a representative sample of the entire cancer cell population [33]. Therefore, the use of human cancer cells taken from individual patients (patient-derived xenograft) and xenotransplanted in zebrafish embryos is an ideal option for the precision oncology or personalized medicine [34]. In fact, the patient-derived xenografts in zebrafish could be a model for in vivo evaluation of drug response and for the identification of the most tailored chemotherapy regimen for each patient [7,15,35]. However, there are few studies reported about the direct transplantation of patient-derived cancer cells in zebrafish, particularly for the colorectal cancer [10,35]. Fior et al. demonstrated the possibility to xenotransplant primary patient cells in zebrafish embryos generating zPDX. Comparing response to chemotherapy and biological therapies between patients and zPDX, these authors showed that zPDXs could be a fast and highly sensitive in vivo model to distinguish the different responses to therapies, revealing the intratumor cancer heterogeneity [10].

However, the use of patient-derived cell samples has some limitations. In fact, the intra-tumor heterogeneity, due to both cell-autonomous (e.g., genomic and epigenomic heterogeneity) and non-cell-autonomous (e.g., stromal heterogeneity) factors, is a crucial aspect that affects the patient specific response to therapy and contributes to the emergence of chemo-resistance [36]. In fact, the gene expression programs can be altered by the cellular interactions with the extracellular matrix, therefore influencing the drive differentiation and altering profoundly the cell behavior. Moreover, stromal cells are components of the tumor microenvironment and the presence of fibroblasts can contribute to the resistance to cytotoxic and targeted therapies [37]. Keeping this in mind, the xenotransplant of both cancer cell and extracellular matrix could be better than the xenotransplantation of patient-derived cells. With this intent, we decided to xenotransplant tumor tissue taken directly from surgical specimen [16]. In fact, in the first part of our co-clinical trial we documented the possibility to directly xenotransplant tissue taken from PDAC and colorectal cancer in zebrafish embryos, and we adopted the relative tumor area (2dpi/1dpi) as primary measure of the study, under the assumption that a statistically significant decrease of this measure with respect to control group (no chemotherapy) is a hallmark of chemotherapy response [12]. Then, to add drugs directly into the fish water [10,12], it was necessary to calculate and validate the equivalent dose human-to-fish that was effective for impaired the growth of both xenotransplanted cancer cell lines and tumor tissue [12]. In our preliminary experiences recently published, we focused our first analysis on PDAC zPDX, due to the aggressiveness of the pancreatic tumor with a higher recurrence rate and a shorter disease free-survival, reporting interesting preliminary results [15,16]. For PDAC zPDX, we compared the results of zPDX tests with data of chemo efficacy published in literature, and we observed that the model reflected the different efficacy of the chemotherapy schemes generally used in the common clinical practice for patients with PDAC [16].

Subsequently, we evaluated the efficacy of our zPDX model also for patients with colorectal cancer. Firstly, to validate the zPDX as a clinical tool to predict the most effective treatment for each patient, we used a linear mixed effect modelling approach. We assumed that the tumor mass could be approximated as a sphere (as demonstrated in Supplementary Methods), and we estimated the tumor volume variation of each zPDX. In all cases, the controls showed an increase of the estimated tumor volume. The aim was to analyze the zPDX to find a stratification with respect to the treatment, allowing the identification of different responses to chemotherapy agents in embryos in 5 days, supporting the applicability of the method for precision medicine.

LMM revealed that zPDXs had a high variable response among treatments. This is consistent with the lack of correspondence between the zPDX and a specific cluster, estimated by Hartigan Wong K-means algorithm. In fact, the cluster analysis revealed an evident patient-specific response in colorectal zPDX treatment group (Figure 3 and Figure 4). Specifically, for each enrolled patient, it was possible to cluster the zPDXs that could benefit from a specific treatment in comparison to the other chemotherapy schemes. The zPDXs of each colorectal cancer patient responded differently to each chemotherapy scheme tested, suggesting the possibility to identify the best chemotherapy scheme patient specifically. The results of this cluster analysis were different from the results that we reported in our zPDXs model for PDAC patients. In fact, in our previous analysis, the PDAC zPDXs of each patient were assigned to the same cluster independently of the treatment tested, theoretically revealing the patients who would have had a better response in general to the chemotherapy. This is not surprising since many PDAC patients have chemo-refractory disease, and just a smaller subset exhibits significant response to chemotherapy. Therefore, this model seems to be able to reflect the biology of different tumors, identifying which PDAC would likely respond to chemotherapy, and which is the best chemotherapy regimen in the case of colorectal cancer.

Moreover, adapting the RECIST criteria as parameter to evaluate the response of zPDX in the colorectal cancer zebrafish trial, we found that the model seems to reflect the different efficacy of the various chemotherapy regimens. In fact, higher response rates were reported with the drugs combination FOLFOXIRI compared with 5-FU alone. Disease progression occurred in 14% in FOLFOXIRI group and 22% in 5-FU group (Figure 5). These results are consistent with literature data that report a higher response rate when the patients are treated with the three main active agents (5-FU, irinotecan, oxaliplatin) constituting the FOLFOXIRI regimen rather than when they are ‘exposed’ to the monotherapy [38,39]. Probably, the percentages of response to 5-FU alone are higher than the real clinical practice. However, colon cancer is a very heterogeneous neoplasm and probably for the number of cases (n = 36) enrolled, in a randomly higher percentage of cases 5-FU resulted more effective than in clinical practice. Nevertheless, we want to point out that the efficacy of the drugs combinations was not dependent on the efficacy of just the single agent 5-FU. In fact, as demonstrated in two explicative case of Figure S3, we detected a statistically significant difference in terms of apoptotic cells in FOLFOXIRI vs. control, but not a statistically significant difference in 5-FU vs. control.

For these reasons, in our opinion this possible bias should not significantly influence the meaning of our study, as 5-FU is the basic drug of every scheme, and there was a concordance between the humans response and zPDX responses described in 75% of cases (6/8 cases) (Figure 6), as suggested also by the Kendall rank correlation test performed by R package Kendall (tau = 0.689, 2-sided p-value = 0.085). Adapting the WHO response evaluation criteria in solid tumors normally used for the 2D assessment of response to chemotherapy, we have already observed that our zPDX model seems to identify PDAC patients who are more likely to respond to chemotherapeutics and that are associated to a favorable survival [15]. Therefore, the results reported for colorectal cancer seem to extend the applicability of our zPDX model also for the prediction of response for patients affected by this type of cancer. Unfortunately, our analysis is based on the data of follow-up obtained in 8 out of 36 patients enrolled in the study, whereas the data reported in our previous analysis on PDAC were supported by data disposable in 16 out of 31 patients enrolled. This could be due to the few events of disease recurrence reported due to a lower biological aggressiveness of colon cancer compared to PDAC, but also due to a lower recurrence rate as well a longer disease-free survival of the patients affected by colorectal cancer compared to PDAC. In this regard, we acknowledge that the number of patients with colorectal cancer ultimately useful for the correlation with the response in zPDXs is still limited, but at the same time, we also underline that, probably due to the novelty of this approach, the number of patients with colorectal cancer enrolled in our co-clinical trial with zPDXs is similar to the very few other published co-clinical studies [7,10,35]. Moreover, as the results reported in our analysis are part of a larger series of the co-clinical trial that also involved patients with PDAC, thus reaching a total of 24 overall cases used in the evaluation of the effectiveness of the model (16 cases of PDAC and 8 cases of colorectal cancer), to the best of our knowledge, our case series becomes one of the largest in literature, and as such we think that our preliminary clinical results could be very promising and meaningful enough to draw some conclusions so far.

Probably, a longer follow-up of the enrolled patients and the increase of the sample size, will allow us to obtain further clinical results to confirm these preliminary data obtained on the prediction of clinical response by our colorectal cancer zPDX model.

## 5. Conclusions

In conclusion, our model seems to be an effective, usable, and inexpensive model for the xenotransplantation of colorectal tumor tissue and for the estimation about the efficacy of the different chemotherapy regimens commonly used in colorectal cancer patients. Our model seems to be promising as the preliminary analysis showed a correlation between the prediction of chemo efficacy reported in zPDXs and the real clinical response to first-line chemotherapy. Moreover, the linear mixed effect model reported as the zPDX treatment response differed among patients, suggesting a patient/tumor specific response.

With regards to the clinical impact of our results, we acknowledge the limitations of immediate application in clinical practice. However, from a prospective point of view, we are also confident that they could give an original contribution to the new frontier of personalized medicine, as our zPDX model might enhance the judgment on the prognosis and the determination of the most appropriate therapy for each patient.

The prospective co-clinical trial is still under way with the intent to extend the analysis of correlation of responses to chemotherapy between the zPDXs and others oncological patients and long-term follow up results are expected to give more conclusive data.

## Figures and Tables

**Table 1:** Colorectal cancer zPDXs were treated with 5-FU, FOLFOX, FOLFIRI, FOLFOXIRI chemotherapy protocols. The drugs in the combinations and their concentrations are reported.

Chemotherapy Protocol	Drugs Combination	Concentration (mg/mL)
5-FU	5-Fluorouracil	0.216
FOLFOX	5-Fluorouracil	0.216
	Folinic acid	0.013
	Oxaliplatin	0.006
FOLFIRI	5-Fluorouracil	0.216
	Folinic acid	0.013
	Irinotecan	0.012
FOLFOXIRI	5-Fluorouracil	0.216
	Folinic acid	0.013
	Oxaliplatin	0.006
	Irinotecan	0.011

**Table 2:** Characteristics of patients with colorectal cancer (*n* = 36).

Characteristics	
Mean age, years ± SD	68.4 ± 12.0 (32–89)
M:F, *n* (%)	22:14 (61.1%:38.9%)
Mean BMI, kg/m2 ± SD	24.4 ± 4.3 (19.9–32.2)
Type of surgical procedure, *n* (%)	
Right hemicolectomy	15 (41.7%)
Anterior rectal resection	9 (25%)
Left flexure resection	4 (11.1%)
Left hemicolectomy	3 (8.3%)
Abdominoperineal resection	2 (5.6%)
Liver metastasis resection	2 (5.6%)
Local recurrence resection	1 (2.8%)
Grade of differentiation, *n* (%)	
G2	20 (55.5%)
G3	16 (45.5%)
Mean tumor dimension, cm	4.7 ± 2.1 (1.2–10.0)
Mean harvest lymph nodes, *n*	21.8 ± 11.8 (6–64)
Mean positive lymph nodes, *n*	3.0 ± 2.8 (0–9)
T status, *n* (%)	
T1	2 (6.1%)
T2	2 (6.1%)
T3	25 (75.8%)
T4	4 (12.0%)
N status, *n* (%)	
N0	8 (24.3%)
N1a	7 (21.2%)
N1b	6 (18.2%)
N2a	5 (15.2%)
N2b	7 (21.2%)
Stage, *n* (%)	
I	3 (8.3%)
IIA	5 (13.9%)
IIB	2 (5.6%)
IIIA	1 (2.8%)
IIIB	13 (36.1%)
IIIC	3 (8.3%)
IVA	7 (19.5%)
IVB	1 (2.8%)
IVC	1 (2.8%)

*M: Male; F: Female; BMI: Body mass index.*

## Data Availability

Dataset and metadata generated and/or analyzed during the current study are available from the corresponding author on request.

## Supplementary Materials

The following are available online at https://www.mdpi.com/article/10.3390/biomedicines10071474/s1, Figure S1: The tumor tissue is xenografted into the perivitelline space of a zebrafish at 2 dpf. Representative images of a zPDX 2 days post xenotransplantation. (A) Bright-field, (B) fluorescence image of human tumor tissue with cell membranes CM-DiI labeled, (C) 8-bit image converted by Image-J, used for the analysis of the tumor area, (D) Representative Hematoxylin & Eosin stained image of a zPDX with xenografted tumor cells (dashed area). Image acquired at 20x magnification; Figure S2: 95% Cis of the difference between fixed (treatments) and random coefficients (zPDXs) estimated by the LMM for 5-FU (A), FOLFOX (B), FOLFIRI (C), and FOLFOXIRI (D) chemotherapy treatments. The dots are the random effect, and the bars represent the 95%CIs of these values. The red dashed line is the mean response to each treatment of the zebrafish embryo populations; Table S1: Fixed effects coefficients, their Standard Error (SE) and *p*-value estimated by LMM; Estimation of the radius; R packages.
