# Supplementary material for: Zebrafish Patient-Derived Xenograft Model to Predict Treatment Outcomes of Colorectal Cancer Patients
Source: Biomedicines. 2022 Jun 22;10(7):1474. doi: 10.3390/biomedicines10071474 (PMC9313122; doi:10.3390/biomedicines10071474)
Supplement: Supplementary file 1 [file biomedicines-10-01474-s001.zip › biomedicines-1770377-supplementary.pdf]

Supplementary Methods

Demonstration that the xenografted tumor mass can be assimilated to a sphere.

The demonstration of the tumor mass circularity is carried out acquiring confocal images of xenografted embryos 2 hpi. The embryos are anesthetized with MS 222 0.16 mg/mL, embedded in a drop of 3% methylcellulose and placed in lumox® dish.

Z-stacks are acquired both on ventral and lateral side for n≥4 embryos from two independent experiment of colon cancer. Then the areas of the tumor from each side are respectively measured, obtaining a ratio of 1.147 ±0.064.

Estimation of the radius at 2hpi and 2dpi

Starting from zPDX area measured at 1dpi and 2dpi, we estimated radius as:

$$r = \sqrt{\frac{Area_{2hpi\ or\ 2dpi}}{\pi}}$$

Apoptotic cells analysis

zPDX were treated with 5-FU and FOLFOXIRI according to Section 2.4 of Materials & Methods and processed for immunofluorescence staining with rabbit anti-human Cleaved Caspase-3 and Hoechst as counterstaining. The quantification of apoptotic cells was performed manually using the Multi point Tool of ImageJ, counting the DiI/Cleaved Caspase double positive with respect to the total number of human cells (DiI-positive cells).

Supplementary Figures

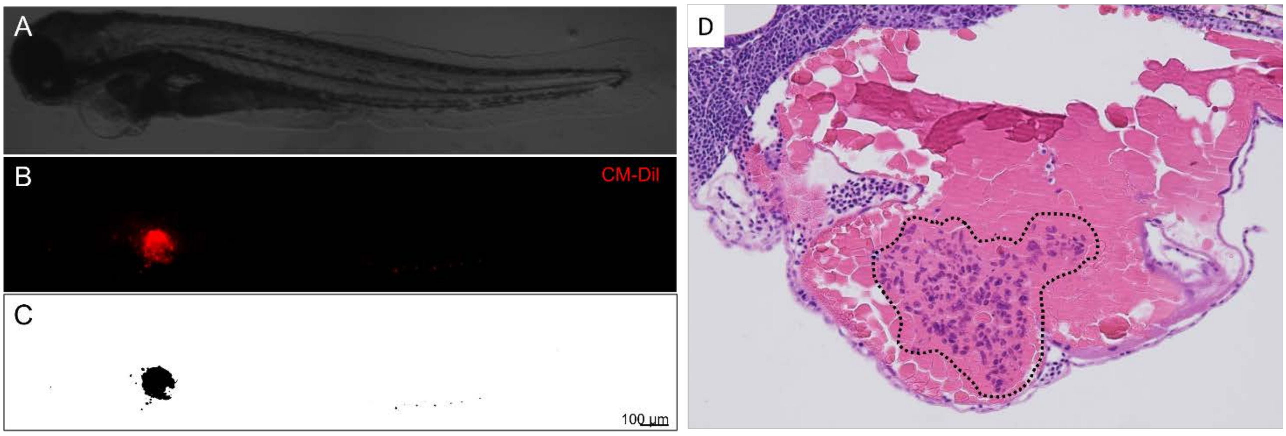

Figure S1 - The tumor tissue is xenografted into the perivitelline space of a zebrafish at 2 dpf. Representative images of a zPDX 2 days post xenotransplantation. (A) Bright-field, (B) fluorescence image of human tumor tissue with cell membranes CM-DiI labeled, (C) 8-bit image converted by Image-J, used for the analysis of the tumor area. (D) Representative Hematoxylin & Eosin stained image of a zPDX with xenografted tumor cells (dashed area). Image acquired at 20x magnification.

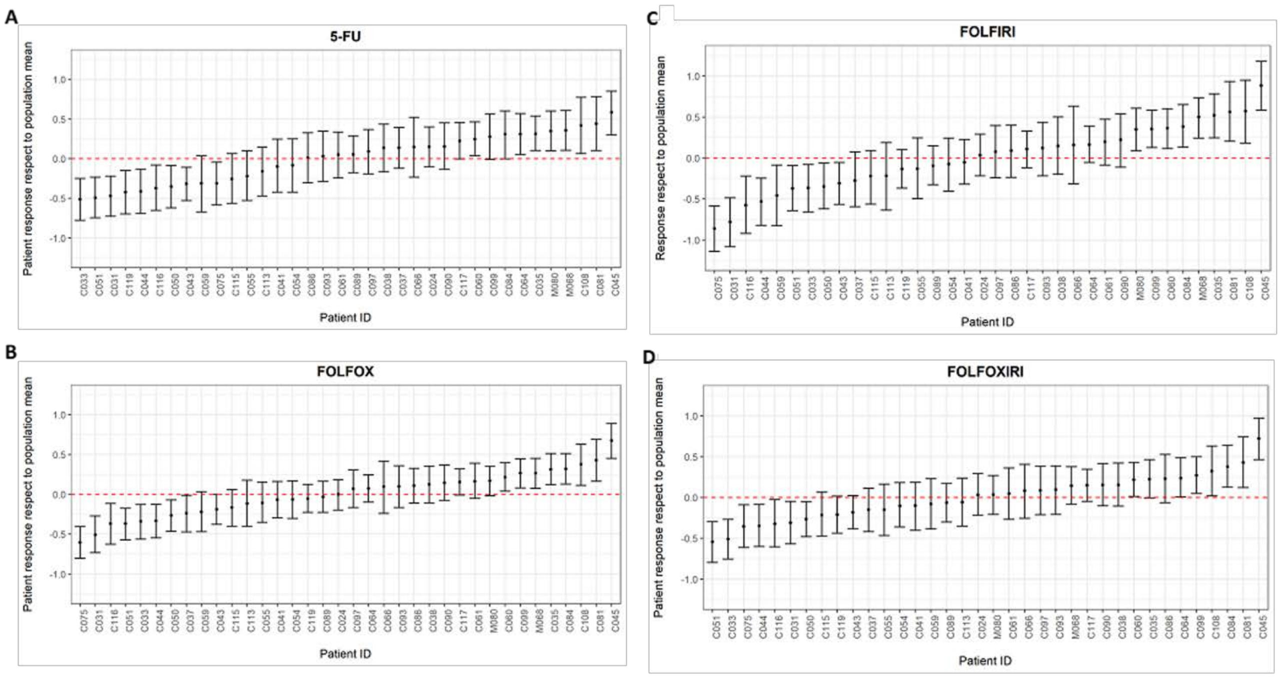

Figure S2. 95% CIs of the difference between fixed (treatments) and random coefficients (zPDXs) estimated by the LMM for 5-FU (A), FOLFOX (B), FOLFIRI (C) and FOLFOXIRI (D) chemotherapy treatments. The dots are the random effect, and the bars represent the 95%CIs of these values. The red dashed line is the mean response to each treatment of the zebrafish embryo populations.

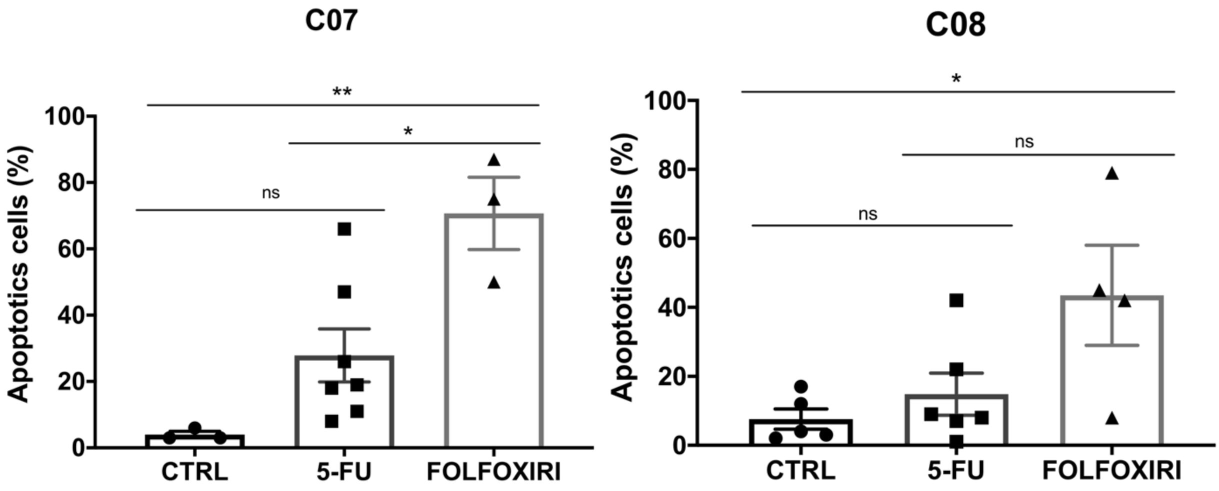

Figure S3. Percentage of human apoptotic cells in zPDX after 5-FU and FOLFOXIRI treatments. Data are expressed as mean ± SEM, n≥3 zPDX for each group. Statistical analysis was performed by Dunnett's multiple comparisons test. *$p < 0.05$, **$p < 0.01$ and ns nonsignificant vs control. C07 and C08 are the patient's identification codes.

Supplementary Table

Table S1. Fixed effects coefficients, their Standard Error (SE) and p-value estimated by LMM.

	Estimate	SE	P-value
**Control**	4.28	0.15	$<2\times10^{-16}$
**5-FU**	-0.19	0.06	4.67×10^{-3}
**FOLFIRI**	-0.28	0.07	4.64×10^{-4}
**FOLFOX**	-0.15	0.06	2.08×10^{-2}
**FOLFOXIRI**	-0.25	0.06	2.80×10^{-4}
**$\log_{10}(r^3)$**	-0.40	0.03	$<2\times10^{-16}$

R packages

1. *car* package: John Fox and Sanford Weisberg (2019). An {R} Companion to Applied Regression. Third Edition. Thousand Oaks CA: Sage. URL: https://socialsciences.mcmaster.ca/jfox/Books/Companion/
2. *dplyr* package: Hadley Wickham. Romain François. Lionel Henry and Kirill Müller (2020). dplyr: A Grammar of Data Manipulation. R package version 1.0.1. https://CRAN.R-project.org/package=dplyr
3. *ggplot2* package: H. Wickham. ggplot2: Elegant Graphics for Data Analysis. Springer-Verlag New York. 2016.
4. *corrplot* package: Taiyun Wei and Viliam Simko (2017). R package "corrplot": Visualization of a Correlation Matrix (Version 0.84). Available from https://github.com/taiyun/corrplot
5. *reshape2* package: Hadley Wickham (2007). Reshaping Data with the reshape Package. Journal of Statistical Software. 21(12). 1-20. URL http://www.jstatsoft.org/v21/i12/.
6. *patchwork* package: Thomas Lin Pedersen (2020). patchwork: The Composer of Plots. R package version 1.1.0. https://CRAN.R-project.org/package=patchwork
7. *readxl* package: Hadley Wickham and Jennifer Bryan (2019). readxl: Read Excel Files. R package version 1.3.1. https://CRAN.R-pro-ject.org/package=readxl
8. *GGally* package: Barret Schloerke. Di Cook. Joseph Larmarange. Francois Briatte. Moritz Marbach. Edwin Thoen. Amos Elberg and Jason Crowley (2020). GGally: Extension to 'ggplot2'. R package version 2.0.0. https://CRAN.R-project.org/package=GGally
9. *broom* package: David Robinson. Alex Hayes and Simon Couch (2020). broom: Convert Statistical Objects into Tidy Tibbles. R package ver-sion 0.7.2. https://CRAN.R-project.org/package=broom
10. *parameters* package: Lüdecke D. Ben-Shachar M. Patil I. Makowski D (2020). "parameters: Extracting. Computing and Exploring the Parameters of Statistical Models using R." _Journal of Open Source Software_. *5*(53). 2445. doi: 10.21105/joss.02445 (URL:https://doi.org/10.21105/joss.02445).
11. *effects* package: John Fox and Sanford Weisberg (2019). An R Companion to Applied Regression. 3rd Edition. Thousand Oaks. CA https://so-cialsciences.mcmaster.ca/jfox/Books/Companion/index.html
John Fox. Sanford Weisberg (2018). Visualizing Fit and Lack of Fit in Complex Regression Models with Predictor Effect Plots and Partial Residuals. Journal of Statistical Software. 87(9). 1-27. URL https://www.jstatsoft.org/article/view/v087i09.
John Fox (2003). Effect Displays in R for Generalised Linear Models. Journal of Statistical Software. 8(15). 1-27. URL https://www.jstatsoft.org/article/view/v008i15.
John Fox, Jangman Hong (2009). Effect Displays in R for Multinomial and Proportional-Odds Logit Models: Extensions to the effects Package. Journal of Statistical Software, 32(1), 1-24. URL https://www.jstatsoft.org/article/view/v032i01.
12. *ggeffect* package: Lüdecke D (2018). "ggeffects: Tidy Data Frames of Marginal Effects from Regression Models." _Journal of Open Source Software_. *3*(26). 772. doi: 10.21105/joss.00772 (URL: https://doi.org/10.21105/joss.00772).
13. *emmeans* package: Russell Lenth (2020). emmeans: Estimated Marginal Means. aka Least-Squares Means. R package ver-sion 1.5.2-1. https://CRAN.R-project.org/package=emmeans
14. *performance* package: Lüdecke. Makowski. Waggoner & Patil (2020). Assessment of Regression Models Performance. CRAN. Available from https://easystats.github.io/performance/
15. *lme4* package: Douglas Bates. Martin Maechler. Ben Bolker. Steve Walker (2015). Fitting Linear Mixed-Effects Models Us-ing lme4. Journal of Statistical Software. 67(1). 1-48.doi:10.18637/jss.v067.i01.
16. *arm* package: Andrew Gelman and Yu-Sung Su (2020). arm: Data Analysis Using Regression and Multilevel/Hierarchical Models. R package version 1.11-2. https://CRAN.R-project.org/package=arm

17. *lmerTest* package: Kuznetsova A. Brockhoff PB. Christensen RHB (2017). “lmerTest Package: Tests in Linear Mixed Effects Models.” _Journal of Statistical Software_. *82*(13). 1-26. doi: 10.18637/jss.v082.i13 (URL: https://doi.org/10.18637/jss.v082.i13)
18. *see* package: Lüdecke. Ben-Shachar. Waggoner & Makowski (2020). Visualisation Toolbox for 'easystats' and Extra Geoms. Themes and Color Palettes for 'ggplot2'. CRAN. Available from https://easystats.github.io/see/
19. *MuMIn* package: Kamil Barton (2020). MuMIn: Multi-Model Inference. R package version 1.43.17. https://CRAN.R-pro-ject.org/package=MuMIn
20. *factoextra* package: Alboukadel Kassambara and Fabian Mundt (2020). factoextra: Extract and Visualize the Results of Mul-tivariate Data Analyses. R package version 1.0.7. https://CRAN.R-project.org/package=factoextra
21. *factoMineR* package: Sebastien Le. Julie Josse. Francois Husson (2008). FactoMineR: An R Package for Multivariate Analy-sis. Journal of Statistical Software. 25(1). 1-18. 10.18637/jss.v025.i01
22. *cluster* package: Maechler. M.. Rousseeuw. P.. Struyf. A.. Hubert. M.. Hornik. K.(2019). cluster: Cluster Analysis Basics and Extensions. R package version 2.1.0.
23. *stats* package: R Core Team (2020). R: A language and environment for statistical computing. R Foundation for Statistical Computing. Vienna. Austria. URL https://www.R-project.org/.
24. *merTools* package: Jared E. Knowles and Carl Frederick (2020). merTools: Tools for Analyzing Mixed Effect Regression Models. R package version 0.5.2. https://CRAN.R-project.org/package=merTools
25. *psych* package: Revelle. W. (2020) psych: Procedures for Personality and Psychological Research. Northwestern Univer-sity. Evanston. Illinois. USA. https://CRAN.R-project.org/package=psych Version = 2.0.9..
26. *classInt* package: Roger Bivand (2020). classInt: Choose Univariate Class Intervals. R package version 0.4-3. https://CRAN.R-project.org/package=classInt
27. *dfidx* package: Yves Croissant (2020). dfidx: Indexed Data Frames. R package version 0.0-3. https://CRAN.R-pro-ject.org/package=dfidx
28. *forcats* package: Hadley Wickham (2020). forcats: Tools for Working with Categorical Variables (Factors). R package ver-sion 0.5.0. https://CRAN.R-project.org/package=forcats
30. *Kendall* package:A.I. McLeod (2011). Kendall: Kendall rank correlation and Mann-Kendall trend test. R package version 2.2. https://CRAN.R-project.org/package=Kendall
